# Thermal effect on the geo-engineering characteristics of a rock salt

**DOI:** 10.1371/journal.pone.0283435

**Published:** 2023-03-23

**Authors:** Nazlı Tunar Özcan

**Affiliations:** Department of Geological Engineering, Hacettepe University, Ankara, Turkey; University of Science and Technology Beijing, CHINA

## Abstract

Rock salt caverns are considered one of the best hosts to store oil, natural gas, radioactive and toxic wastes due to their low permeability, self-healing characteristics and wide distribution on the Earth. Stored nuclear waste in rock salts will radiate for many years. Therefore, the thermal energy and also temperature in the host environment will increase depending on time. In this study, P-wave velocity (V_p_), Brazilian tensile strength (σ_t_), uniaxial compression strength (σ_c_) of Çankırı rock salt were investigated under different temperatures ranging from 20°C to 250°C since the temperature is a factor that causes changes in some physical and geo-mechanical properties of rocks. The acoustic emission technique was utilized during uniaxial compression strength tests, to monitor the crack accumulation. Additionally, X-ray micro-computed tomography technique was employed to observe the microstructure and determine the porosity of rock salt samples depending on the temperature. The V_p_ and the σ_t_ of Çankırı rock salt decrease with increasing temperatures of samples whereas the σ_c_ increases. The ductility of rock salt tends to increase with augmented temperature and the axial strain at the ultimate stress level is 2.96% at 20°C whereas it reaches up to 6.29% at 250°C. The AE activity of rock salt generates at the early stages of loading and AE count prominently increases with the increasing temperature of samples. Therefore, the stress levels of crack initiation (σ_i_) and crack damage (σ_cd_) thresholds were reached earlier than the previous one with each temperature increment. According to X-ray micro-CT images of rock salts, the number of cracks increased markedly in thermally treated rock salt samples and therewith the porosity increases from 1.12% to 2.73% with an increase in temperature from 50°C to 250°C.

## Introduction

Rock salt is recently employed in the storage of petroleum and natural gas as well as nuclear waste repositories due to its very low permeability, visco-plastic deformation behavior and self-healing characteristics. Using rock salt as a host rock has economic importance in terms of enabling the storage of resources that will meet energy needs. It also provides crucial worldwide environmental safety by isolating hazardous wastes.

Nuclear wastes continue to radiate for many years due to long radioactive half-life. Thereby, the thermal energy in the hosting medium increases over time. Soppe et al. [1] determined that the temperature of the rock salt in the close vicinity of the waste container gets warmed up to 165°C. Similarly, the temperature of a rock salt storage cavern can increase up to 140°C during the injection and withdrawal processes of natural gas in relation to the injection rate, pressure, and maximum storage volume [2, 3]. Consequently, it is essential to reveal the effects of temperature increase on the geo-mechanical and physical properties of surrounding rock salt. There are many studies [e.g. 4–12] on the mechanical properties and long term behaviour of rock salt whereas the studies investigating the effect of temperature on these properties are limited. In the previous studies, there are three different types of testing procedures encountered: (1) heating the samples to certain temperatures through special heating apparatus implemented to experimental setup, (2) heating the samples in an oven and performing the test immediately after, (3) by using the samples cooled to room temperature after heating (thermally treated sample). Liang et al. [13] found that uniaxial compressive strength (σ_c_) and shear strength parameters increase, and ultrasonic velocity decreases with increasing temperature (20°C – 240°C) of thenardite salt rock. Liang et al (2006) determined that the thermal effect has significant impacts on the properties which control the repository quality of rock salt and suggested that more research is required on the physical and geo-mechanical characteristics of rock salt at high temperatures. Li et al. [14] conducted thermo-mechanical coupled triaxial compression tests on two kinds of pure rock salt and impure (interbedded) rock salt specimens at temperatures 53°C and 65°C. The researchers indicated that the σ_c_ and failure mode of rock salt is sensitive to temperature changes and the σ_c_ decreases with rising temperature. Li et al. [14] pointed out that rock salt behaves from brittle to ductile with increasing temperature. Sriapai et al. [15] revealed that the σ_c_ and tensile strength (σ_t_) of the thermally treated (0°C – 194°C) cubical rock salt samples decrease linearly as treatment temperature increases. One can be seen that there are inconsistencies in the results of the previous studies due to differences in the shape of specimens, rock salt types (pure, interbedded, one crystalline, polycrystalline), heating processes and testing procedures.

The acoustic emission (AE) measurement is a robust and well-known tool for monitoring the microcrack accumulation in rocks during the loading stage [16–22]. The AE in geological materials is defined as a transient elastic wave generated from the rapid release of unit deformation energy during micro-fractures and fractures (Hardy, 1972). It is possible to get information about the properties of the medium and deformation mechanisms by AE measurements [23–25]. Another non-destructive technique used to observe microcrack propagation is the combination of X-ray computed tomography (CT) and digital image processing methods. X-ray CT is a popular tool to provide high-resolution reconstructed three-dimensional images of geologic materials by X-ray beam attenuation [26–28].

The aim of this study is to investigate the thermal effect on the σ_c_, σ_t_, V_p_, and crack accumulation of Çankırı rock salt for temperatures ranging between 20°C and 250°C. Rock salt samples at room temperature and the heated samples at temperatures of 50°C, 100°C, 150°C, 200°C and 250°C were subjected to uniaxial compression and Brazilian tensile tests. The AE technique was utilized while applying the uniaxial compression tests to examine the microcrack accumulation resulting from the thermal effect of rock salt. The stress levels of crack initiation (σ_i_) and crack damage (σ_cd_) thresholds were determined and the thermal effect on these stress levels was evaluated. In addition, the changes in microstructure and porosity of thermally treated at different temperatures rock salt samples demonstrated by using X-ray micro-CT technique. The novelty of this study is utilizing the non-destructive methods both the AE and the X-ray micro-CT in the assessment of the thermal effect on rock salt for the first time.

## Description of Çankırı rock salt

**Fig 1:**
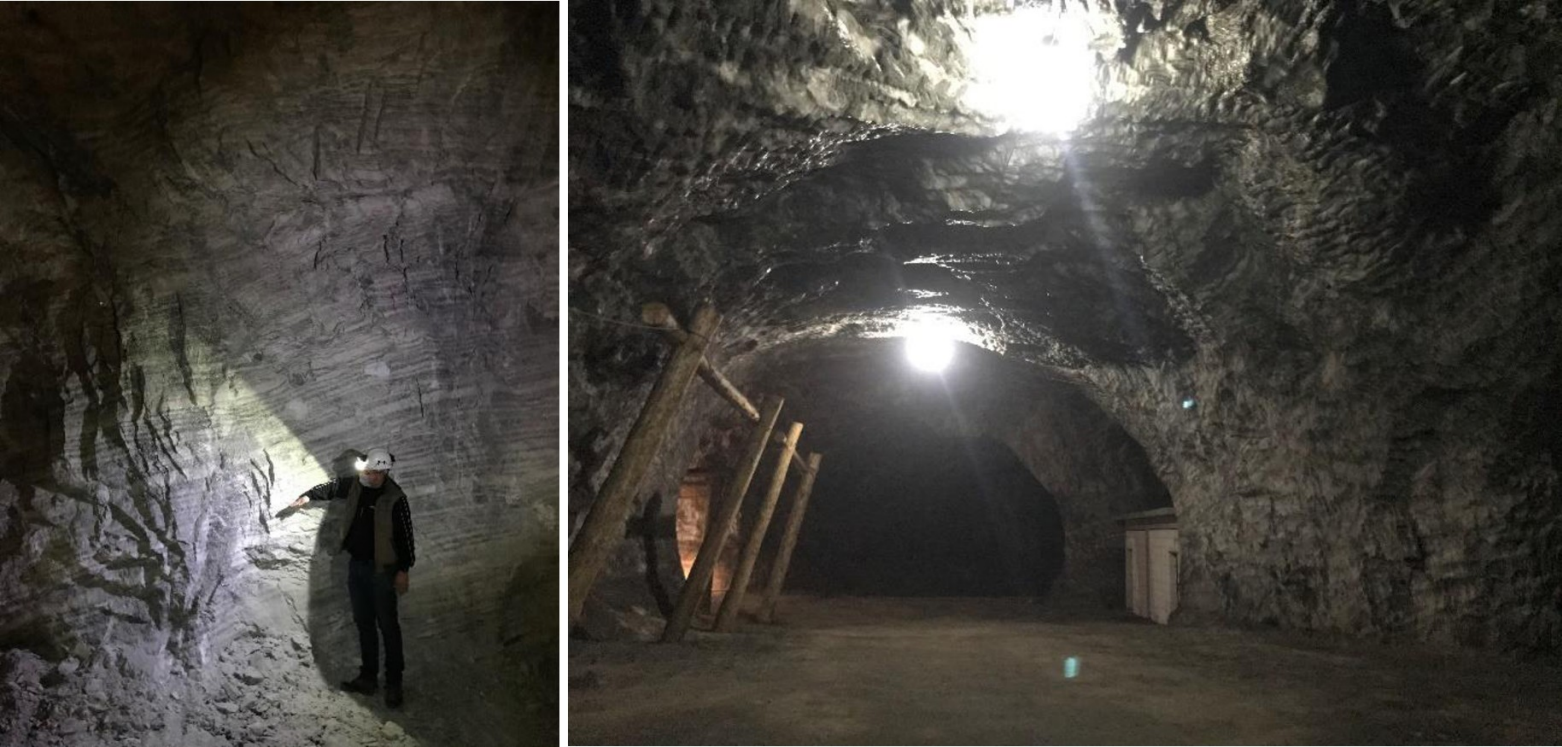
Some views from Çankırı rock salt mine.

Due to the droughts experienced in the Eocene, Oligocene and Miocene periods, salt formations have been widely deposited in Turkey. More than 30 salt beds connected to each other lie down from Çankırı (Central Anatolia) to Iran [29]. Çankırı rock salt mine (Central Anatolia, Türkiye) is thought to have been operated for 5,000 years since the Hittite period (3000 BC) [30]. Rock salts were deposited in the Oligo-Miocene-aged Gypsum Series and the mine has the largest rock salt reserves of Türkiye with 812 million tons [29]. The rock salt formation was tectonically deformed and folded. The mine is operated by an underground room and pillar method (Fig 1). Considering Çankırı rock salt underground mines could be a potential high level nuclear waste repository area, Çankırı rock salt is chosen as the material of this study. Rock salt blocks were extracted from Çankırı rock salt mine.

**Fig 2:**
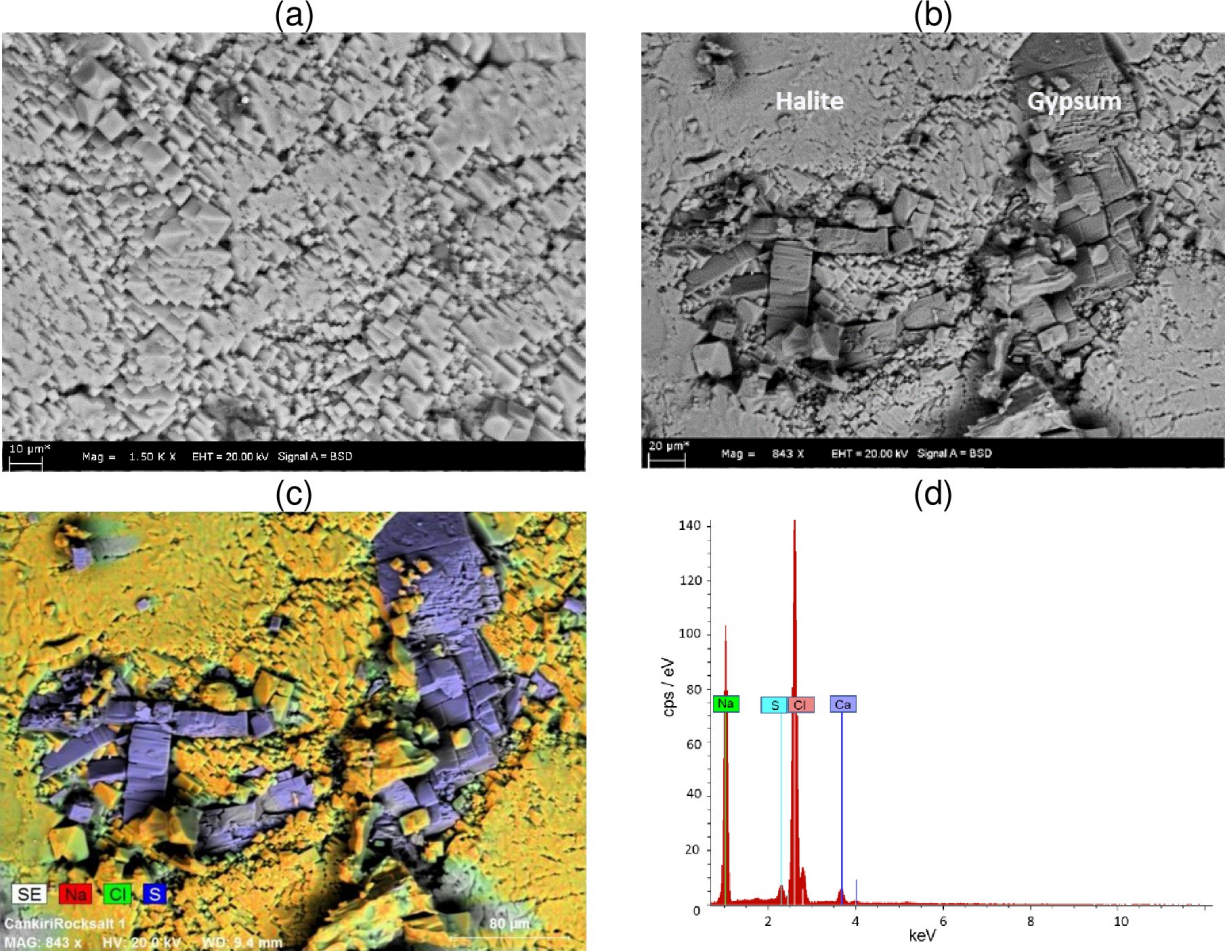
(a, b) Scanning electron microscopy micrographs and (c, d) the chemical analysis by energy dispersive spectrometry of the Çankırı rock salt sample.

The color of the rock salt is not uniform and it contains white colored and mainly isometric cubical shaped halite crystals with some grey colored parts. The rock salt structure is coarse grained and the texture of it is massive. Çankırı rock salt is not properly pure, with nearly 5% clayey insoluble components. The microstructure of the Çankırı rock salt was examined on a Zeiss EVO 50 EP scanning electron microscope (SEM) with 40-Pa to 70-Pa vacuum and 10 mm distance. Cubicle NaCl crystals (Fig 2A) and gypsum minerals (Fig 2B) are observed in SEM images. Additionally, the chemical composition of the rock salt was also determined by energy dispersive spectrometry (EDS) attached to the SEM, since some dark colored parts were observed in samples. With EDS, the amount of the elements were determined by using the area ratio of characteristic X-rays. Accordingly, the rock salt sample is composed of 49.4 wt.% Cl, 45.2 wt.% Na_2_O, 3.5 wt.% CaO and 1.86 wt.% S (Fig 2C and 2D). Detection of large amounts of Cl and Na_2_O in EDS indicates the presence of predominantly NaCl and small amounts of S and CaO are the indicators of gypsum. These results are consistent with the SEM images, and the coarse crystalline part represented by the purple colored in Figure **c is gypsum minerals.

## Laboratory studies and tests results

**Fig 3:**
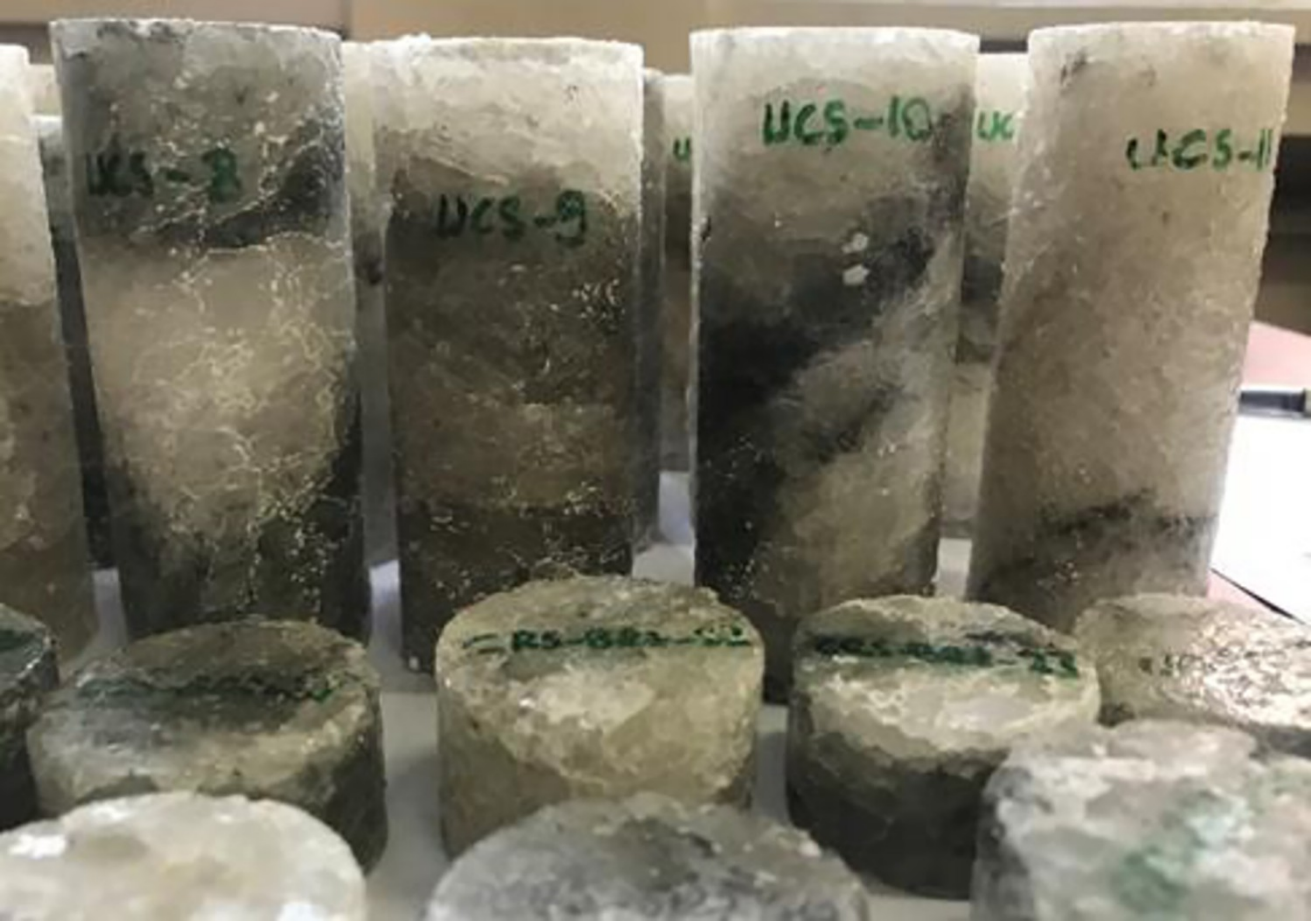
Some rock salt core samples prepared for uniaxial compression and Brazilian tensile tests.

**Fig 4:**
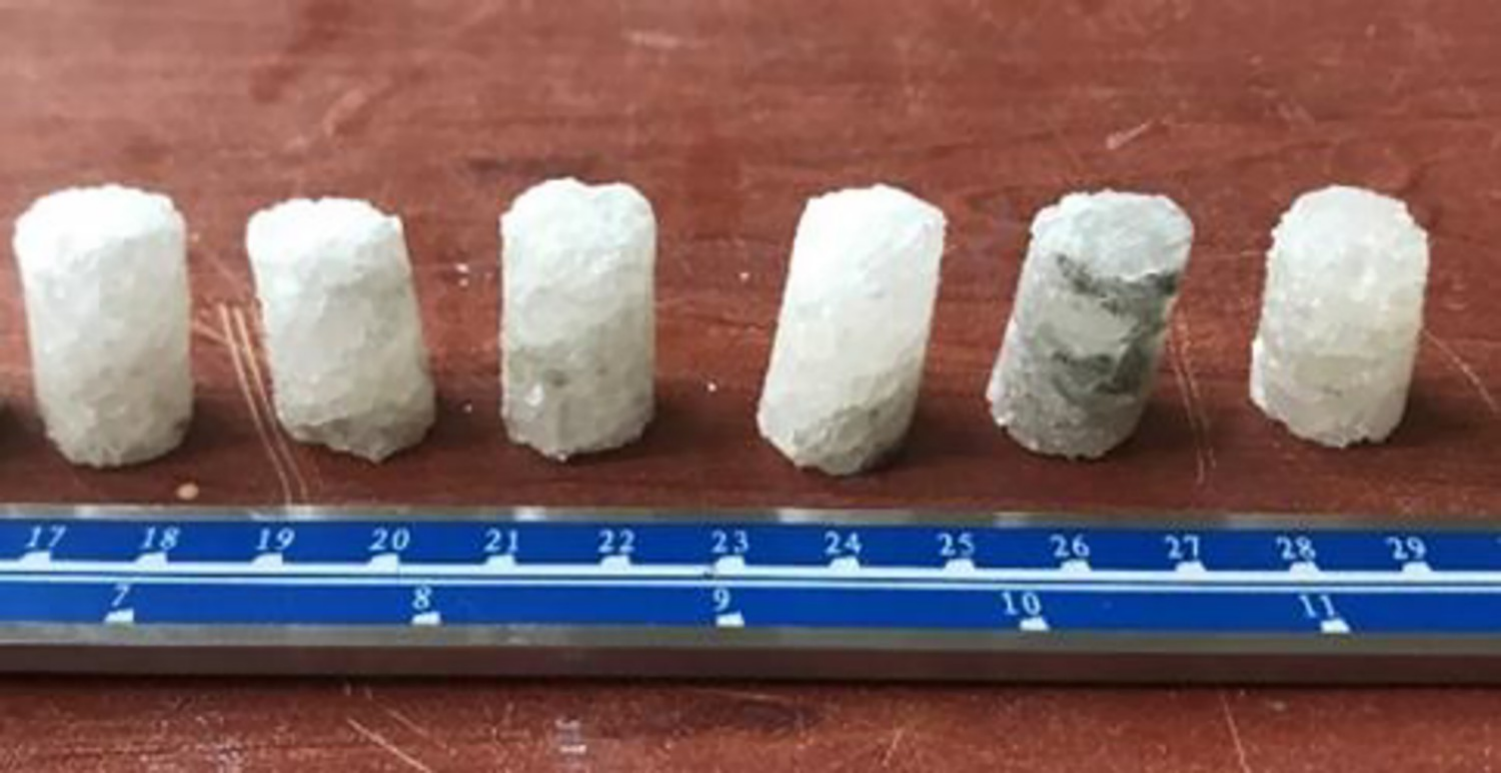
Samples used in X-ray micro computed tomography.

A series of laboratory tests were performed to investigate of the thermal effect on Çankırı Rock Salt. In this context, core samples with a length/diameter ratio of 2.5 to 3 were prepared by using diamond tipped rock bit in accordance with the ISRM [31] standards in the laboratory. To prevent rock salt from dissolution, a very small amount of water was used while coring. Some dissolved and deformed rock salt core samples were eliminated and only those samples that preserve their uniform cylindrical core shape were subjected to tests. A total of 30 core specimens and 25 Brazilian test specimens were extracted (Fig 3). Additionally, 6 rock salt samples with a maximum diameter of 25 mm and a height of 30 mm were prepared for the X-ray micro-CT experiments (Fig 4). Evaporitic rocks generally do not have small-scale planes of weakness at the test sample size, such as thin-bedded sedimentary rocks or metamorphic rocks with schistosity. Care was taken to ensure that there was no visible bedding plane or any discontinuity plane in the sample size. Thereby, Çankırı rock salt samples were considered homogeneous in laboratory scale.

**Fig 5:**
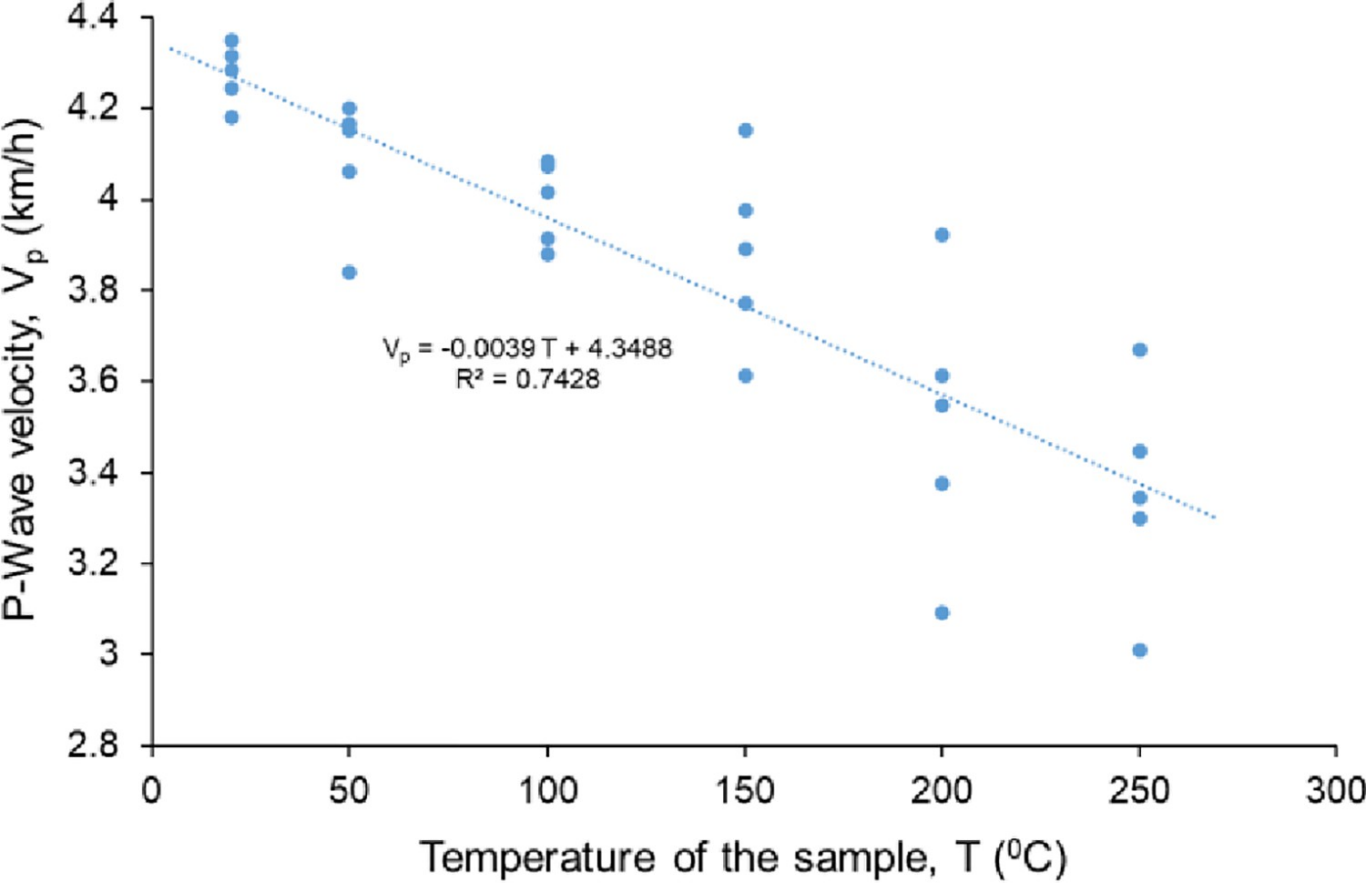
P-wave velocities of rock salt samples at different temperatures.

The dry unit weight of rock salt samples ranges between 19.6 kN/m^3^ and 21.0 kN/m^3^. Five core specimens for each temperature level were heated to the temperatures of 20°C, 50°C, 100°C, 150°C, 200°C and 250°C in the oven for 48 hours. The V_p_ of the samples was measured before the samples were heated and immediately after removing them from the oven. The V_p_ values range from 3.86 km/h to 4.51 km/h and from 3.00 km/h to 4.34 km/h before and after heating, respectively (Table 1). There is a negatively proportional linear relationship between V_p_ and temperature as seen in Fig 5.

**Table 1:** The P-wave velocity, Brazilian tensile strength, and uniaxial compressive strength test results of the rock salt.

Sample No	T (°C)	P-wave velocity (km/s)	σ_t_ (MPa)	σ_c_ (MPa)
CRS_UCS_1	20	4.28	2.06	23.85
CRS_UCS_2	20	4.31	2.07	23.71
CRS_UCS_3	20	4.18	2.01	23.47
CRS_UCS_4	20	4.24	2.24	24.72
CRS_UCS_5	20	4.35	2.4	22.71
CRS_UCS_6	50	4.17	2.15	24.72
CRS_UCS_7	50	4.15	1.73	23.10
CRS_UCS_8	50	4.06	2.13	24.62
CRS_UCS_9	50	3.84	1.99	24.50
CRS_UCS_10	50	4.20	2.07	25.71
CRS_UCS_11	100	4.08	1.8	25.28
CRS_UCS_12	100	3.91	1.81	25.90
CRS_UCS_13	100	4.07	1.79	25.42
CRS_UCS_14	100	4.01	1.64	24.12
CRS_UCS_15	100	3.88	1.98	24.95
CRS_UCS_16	150	3.98	1.83	27.07
CRS_UCS_17	150	3.61	1.79	25.30
CRS_UCS_18	150	3.89	1.59	24.24
CRS_UCS_19	150	3.77	1.5	26.60
CRS_UCS_20	150	4.15	1.61	25.70
CRS_UCS_21	200	3.38	-	26.58
CRS_UCS_22	200	3.09	-	29.10
CRS_UCS_23	200	3.92	-	26.00
CRS_UCS_24	200	3.55	-	25.73
CRS_UCS_25	200	3.61	-	27.75
CRS_UCS_26	250	3.01	1.82	28.16
CRS_UCS_27	250	3.30	1.44	28.18
CRS_UCS_28	250	3.67	1.59	26.46
CRS_UCS_29	250	3.45	1.65	27.57
CRS_UCS_30	250	3.34	1.56	28.45

**Fig 6:**
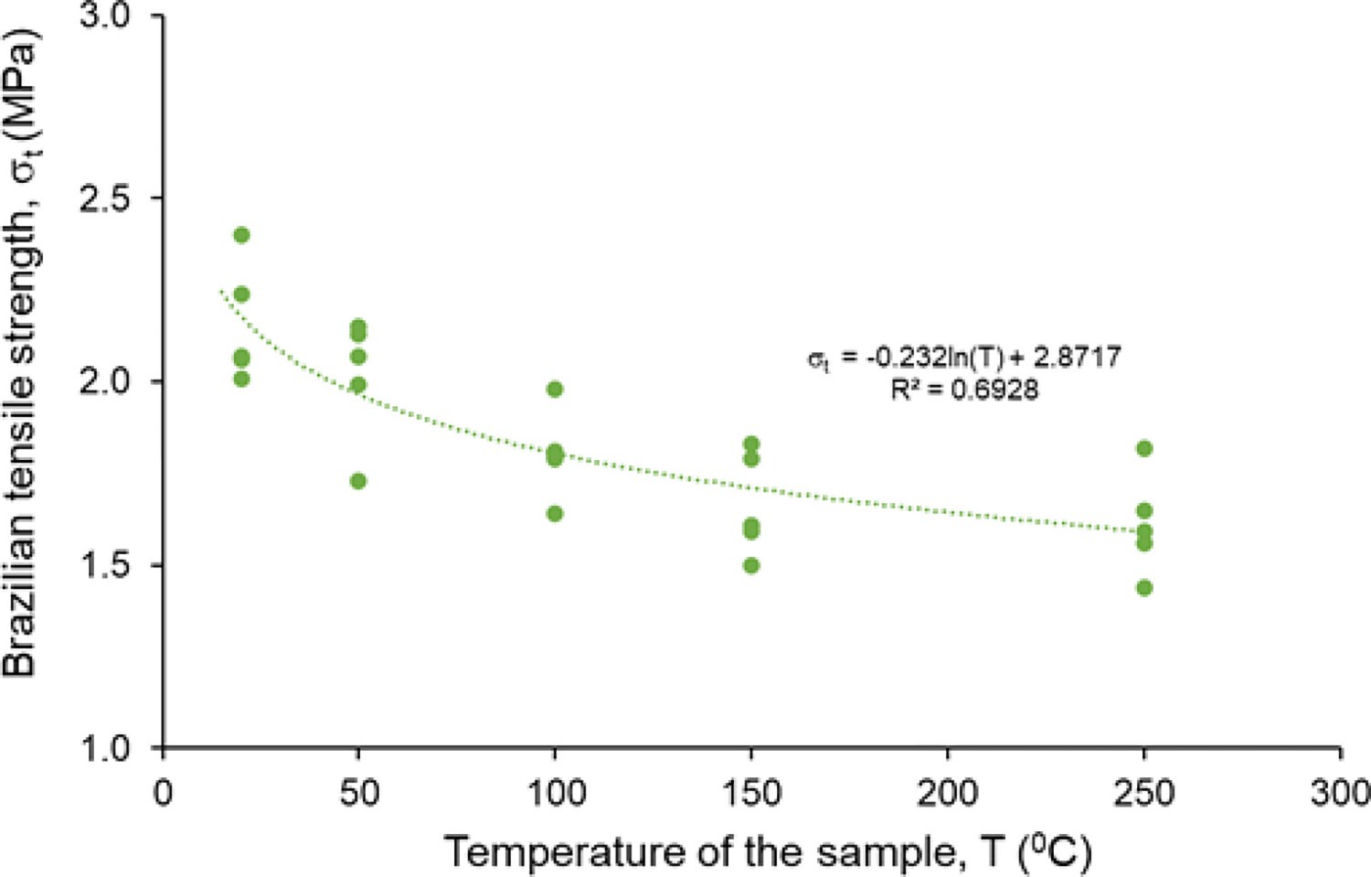
The variation of Brazilian tensile strength of Çankırı rock salt at different temperatures.

The Brazilian tensile strength of rock salt was determined at different temperatures. The σ_t_ of non-heated rock salt samples ranges between 2.01 MPa and 2.40 MPa at room temperature (20°C). The average σ_t_ is decreased from 2.16 MPa to 1.61 MPa with the increasing temperature up to 250°C (see Table 1). The σ_t_ values of samples show a non-linear decreasing trend as the temperature of the test sample increases as seen in Fig 6.

**Fig 7:**
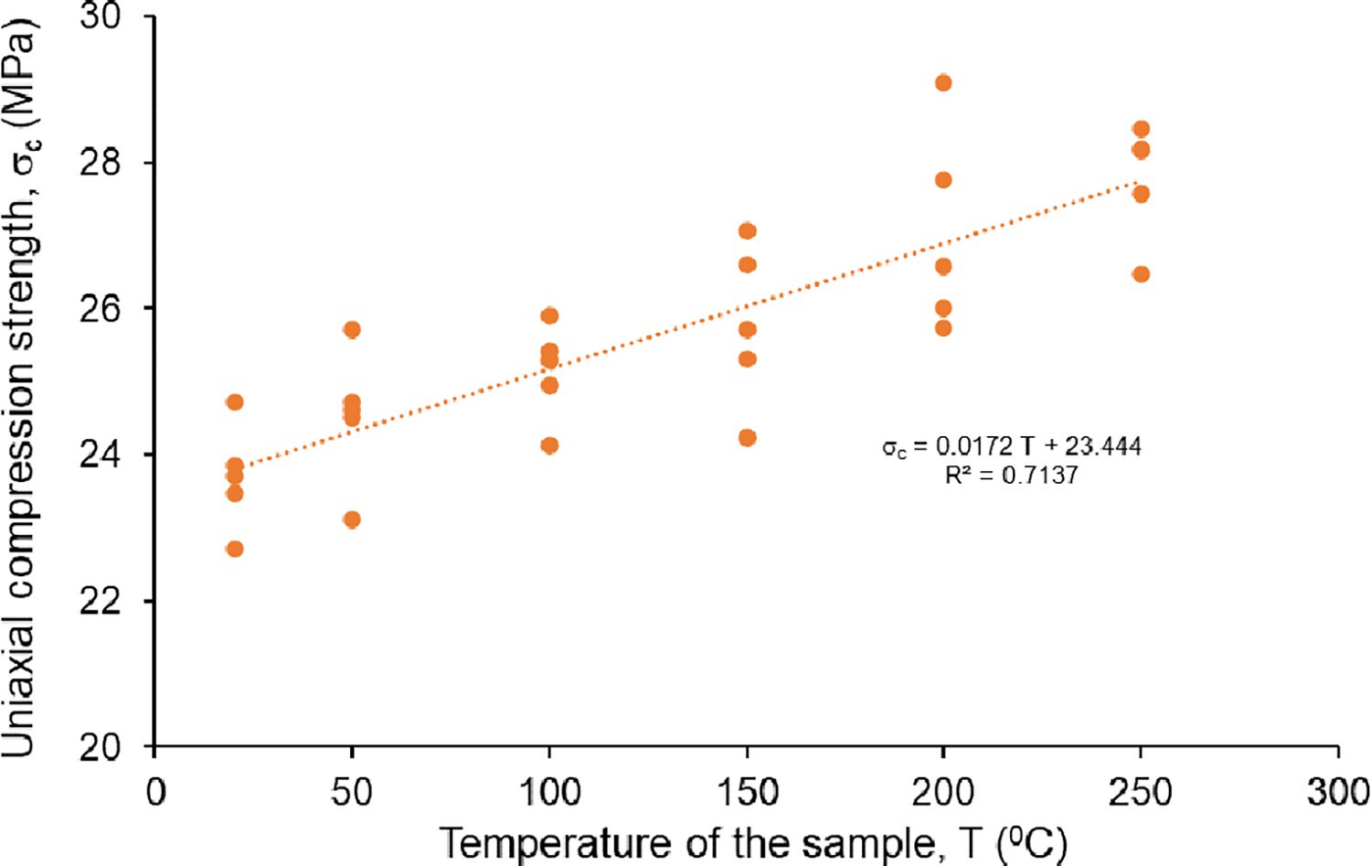
The variation of the ↑_c_ of the rock salt at different temperatures.

**Fig 8:**
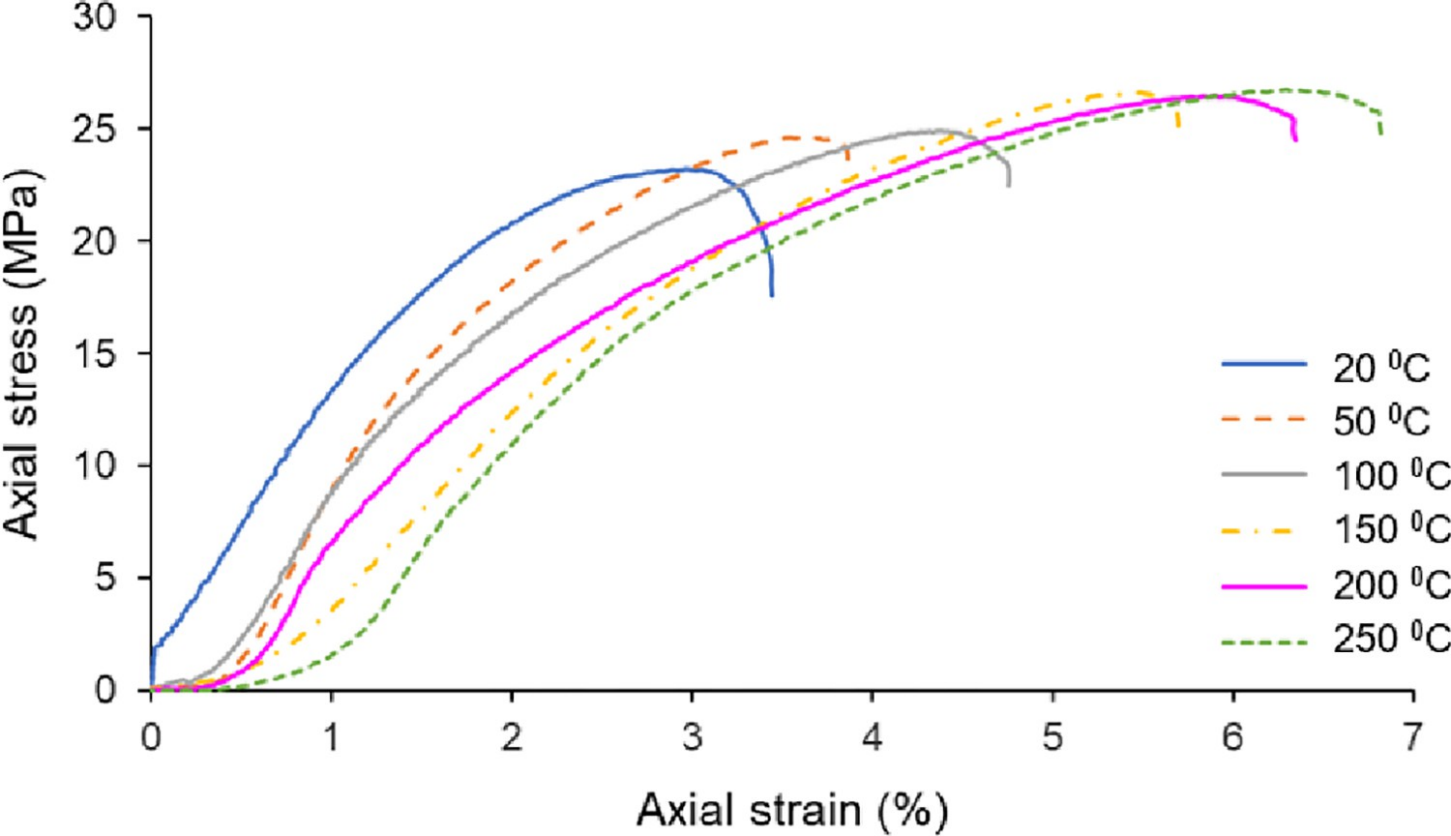
Axial stress and axial strain relationships for various temperatures.

The uniaxial compression tests were performed by using a strain-controlled test device with a loading rate of 0.7 mm/min. The rock salt specimens failed within 6 to 9 minutes after the initial load was applied. The uniaxial compression strength of non-heated Çankırı rock salt varies between 22.7 MPa and 24.7 MPa (see Table 1). The low standard deviation (0.65) of UCS values of non-treated natural samples is also an indicator of the homogeneity of the test samples. The samples heated up to 50°C, 100°C, 150°C, 200°C and 250°C were subjected to uniaxial compressive strength tests immediately after coming out of the oven. The variation of the σ_c_ values with the heating temperatures of the samples is depicted in Fig 7. Accordingly, it was determined that the average σ_c_ of the samples linearly increased from 23.7 MPa to 27.7 MPa with the temperature increases. The stress-strain curves of all samples at different temperatures are also presented together in the graph given in Fig 8. The effect of temperature on rock salt behavior under axial stresses is clearly demonstrated in this graph. It is determined from the stress-strain curves that the amount of strain for the same stress levels increased with the increasing temperature of the samples and therefore the rock salt shows more ductile behavior with the effect of temperature increments. One can see from this figure that decreasing slope of stress-strain curves indicates brittle to ductile behavior transition with increasing temperature.

**Fig 9:**
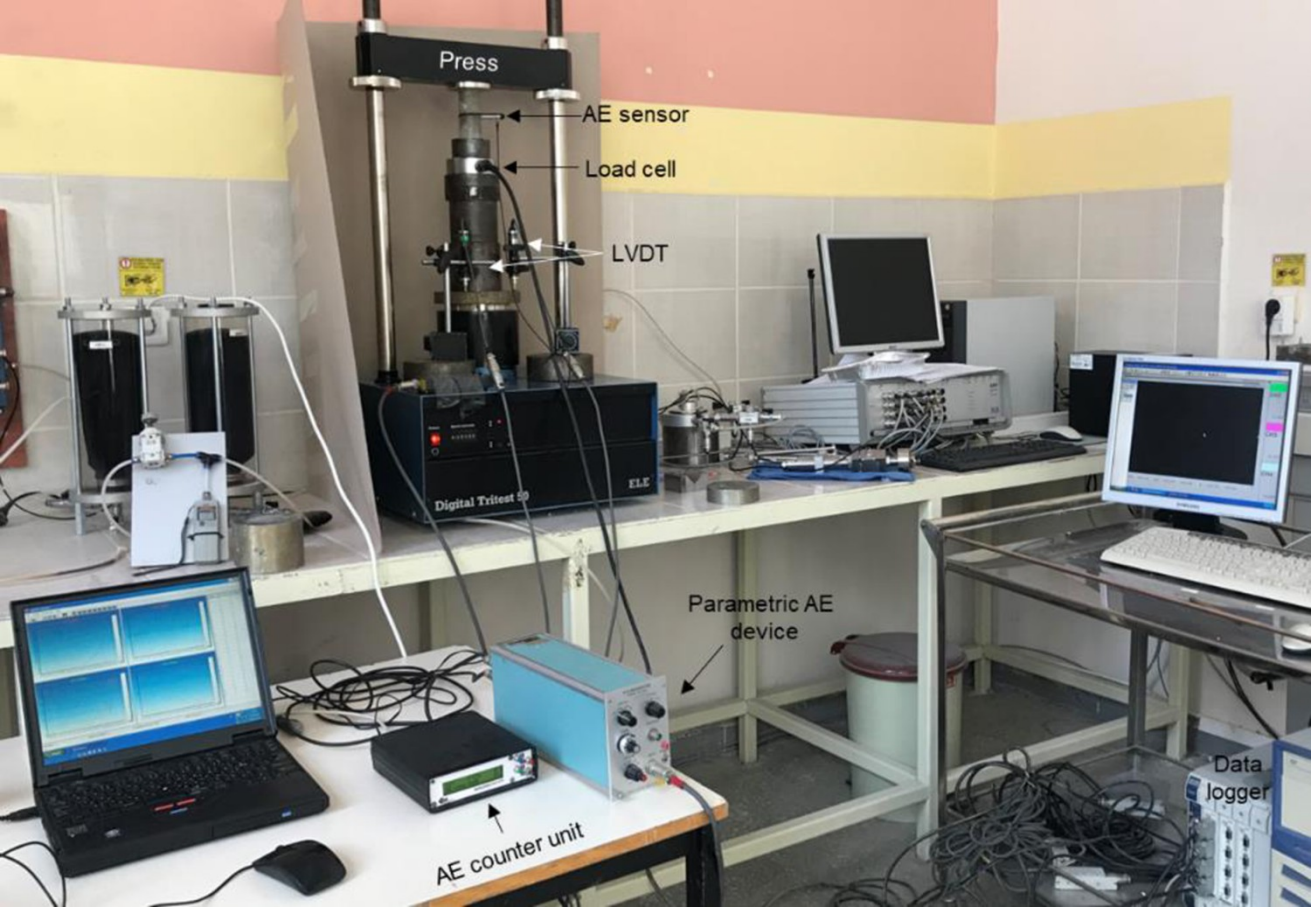
The experimental setup of uniaxial compressive strength test and AE monitoring.

**Fig 10:**
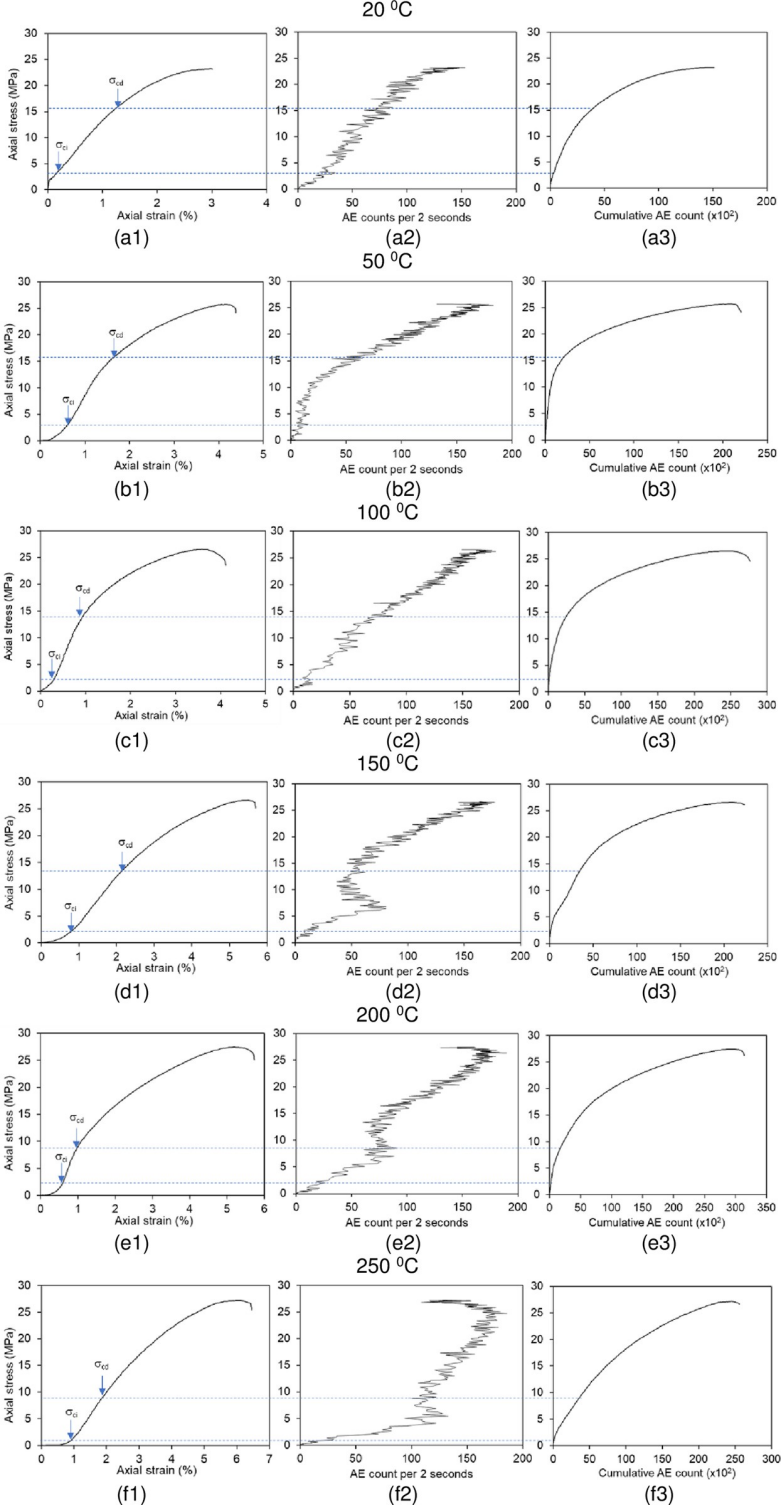
Stress strain curves (from a1 to f1), stress—AE count plots (from a2 to f2) and stress—cumulative AE count plots (from a3 to f3) for the sample temperatures of 20°C, 50°C, 100°C, 150°C, 200°C and 250°C.

To monitor the thermal effect on microcrack accumulation of the rock salt, the AE measurements were recorded while uniaxial compression strength tests, simultaneously. The time dependent AE counts were recorded by a portable parametric AE system. The system consists of a transducer including piezoelectric material with a resonant frequency of 150 kHz, a preamplifier with 20 dB and 40 dB amplification, and a main unit. The signal detection frequency range of the transducer is 10 kHz to 400 kHz. The main unit is equipped with a post-amplifier with an amplification capacity in the range of 10 dB– 40 dB, high and low pass filters and a threshold signal detection unit. The whole system setup used in the tests is shown in Fig 9. AE count and AE ring-down count values were recorded during the tests. Then, “Axial stress” vs. “axial strain”, “axial stress” vs. “AE count per 2 sec.” and “axial stress” vs. “cumulative AE count” graphs for each sample temperature are plotted according to uniaxial compression test and AE measurements (Fig 10). As can be seen from the “axial stress” vs. “AE count per 2 sec.” and “axial stress” vs. “cumulative AE count” graphs, the AE activity starts rapidly from the early stages of loading and increases continuously until the peak stress is reached. In addition, cumulative AE count is properly increased with increasing axial stress levels. The AE counts recorded at the same stress level prominently increase as the temperature of samples increases (Fig 10A2–10F2). This result shows that temperature has a strong effect on the AE characteristics of rock salt regarding ductile behavior increasing with temperature. The stress levels of crack initiation (σ_ci_) and the crack damage (σ_cd_) were also determined from stress-strain curves and these stress levels were verified with stress-AE count and stress-cumulative AE count plots for each temperature of sample (Table 2). The determination of σ_ci_ and σ_cd_ thresholds are made in line with the methods followed by Brace [32], Bieniawski [33], Ohnaka and Mogi [34], Martin and Chandler [35], Eberthardt et al. [36] and Diederichs et al. [17] and shown in Fig 10. While the σ_ci_ and σ_cd_ thresholds of the non-heated samples are 3.9 MPa (16.8% of the peak stress) and 15.6 MPa (67.2% of the peak stress), the σ_ci_ and σ_cd_ thresholds of the samples heated up to 250°C decreased dramatically to 0.98 MPa (3.6% of the peak stress) and 9 MPa (33.1% of the peak stress). Eventually, the cracking processes of rock salt samples initiates at the earlier phase of the loading with the increasing temperature.

**Table 2:** The F073_ci_ and F073_cd_ values of samples with different temperatures.

T (°C)	σ_ci_ (MPa)	σ_cd_ (MPa)
20	3.9	15.6
50	3.2	15.2
100	2.12	14.14
150	1.73	13.35
200	2.2	8.5
250	0.98	9

**Fig 11:**
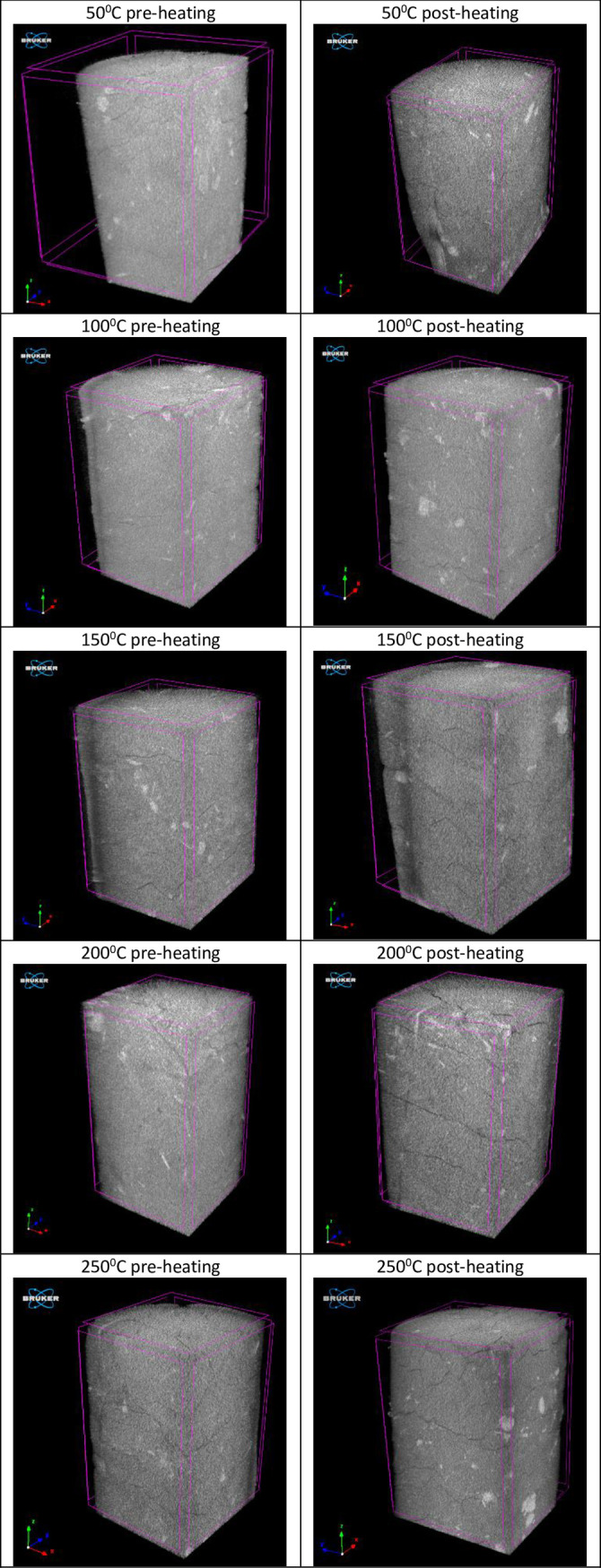
X-ray micro computed scans of the pre-heated and heated rock salt samples.

Additionally, X-ray micro-CT technique was utilized to observe the changes in microstructure and porosity of thermally treated rock salt samples at different temperatures. The X-ray scans of five rock salt samples were made by using Bruker SkyScan 1272 high-resolution scanner before and after thermal treatment. To generate a three-dimensional (3D) representation of rock salt samples, the two-dimensional (2D) image set obtained from X-ray scans was reconstructed using InstaRecon’s algorithm. Data processing and analysis such as segmentation, thresholding for noise reduction, calculation of the volume of interest (VOI) and object volume (V) were performed with CTVol, CTAn and CTVox software. Reconstructed 3D images of rock salt samples before and after thermal treatment with temperatures 50°C, 100°C, 150°C, 200°C and 250°C are demonstrated in Fig 11. At 50°C, some tenuous microcracks were observed in the X-ray micro-CT images of the rock salt sample. When heating to 100°C, the grain boundaries became sensitive to rupture and microcracks propagated visibly through the sample. It is observed that more cracks developed, and the existing cracks expanded with the increasing treatment temperature from the rock salt samples which come out of the oven. It is clearly seen from Fig 11, that thermally treated samples contain more cracks formed as a result of plastic deformations arising from thermal effect. Besides, the number of cracks is augmented as the treatment temperature increases.

Thanks to the VOI and the V values calculated from X-Ray micro-CT scans, the porosities of non-treated and thermally treated samples were calculated from the Eq 1 and the results is given in Table 3.

n = (TV/V)*100 (1)

Where;

TV: Total volume of interest (mm^3^)

V: Object volume (mm^3^)

n: Porosity or percent object volume (%)

**Table 3:** Porosity parameters of rock salt calculated from X-ray micro-CT analysis.

Sample No	Non-treated			Treatment temperature (°C)	Thermally treated			Increment (%)
	VOI_1_ (mm3)	V_1_ (mm3)	V_1_/TV_1_ (%)		VOI_2_ (mm3)	V_2_ (mm3)	V_2_/TV_2_ (%)	
CRS_1	1501.7	30.48	2.03	50	1264.5	28.83	2.28	10.96
CRS_3	1792	24.91	1.39	100	1311.5	22.56	1.72	19.19
CRS_7	1277.7	13.93	1.09	150	1969.7	37.42	1.90	42.63
CRS_8	1507.4	15.98	1.06	200	1815.2	44.84	2.47	57.09
CRS_5	1569.1	17.57	1.12	250	2048.8	55.93	2.73	58.97

**VOI**_**1**_: total volume of interest of non-treated rock salt sample, **V**_**1**_: object volume of non-treated rock salt sample

**V**_**1**_**/TV**_**1**_^:^ Porosity of non-treated rock salt sample, **VOI**_**2**_: total volume of interest of thermally treated rock salt sample, **V**_**2**_: object volume of thermally treated rock salt sample, **V**_**2**_**/TV**_**2**_^:^ Porosity of thermally treated rock salt sample.

**Fig 12:**
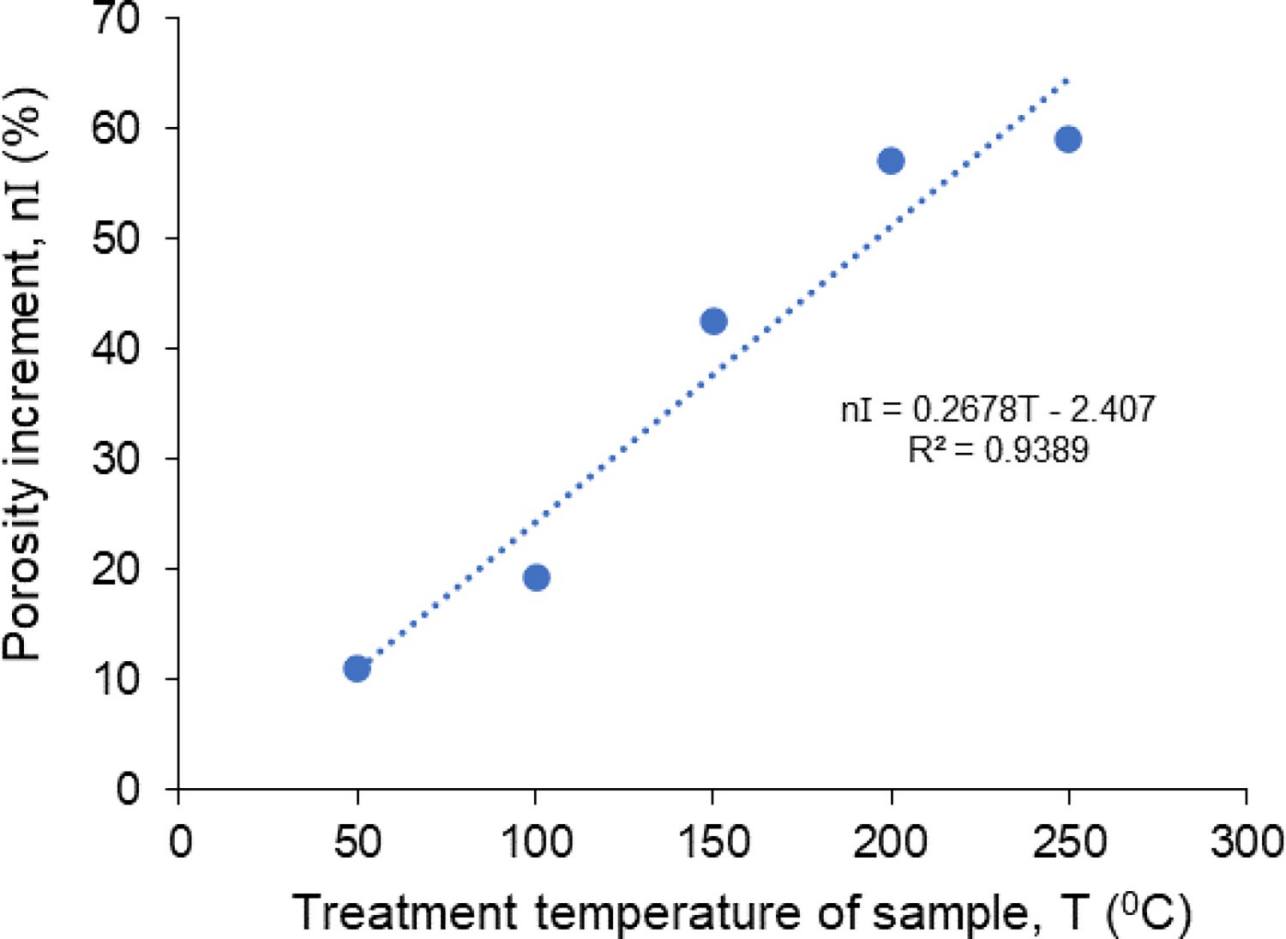
The porosity increments with increasing treatment temperatures of rock salt samples.

The porosities of non-treated samples and thermally treated samples vary between 1.06% - 2.03% and 1.72% - 2.73%, respectively. The increment percentages of porosity changing with the temperatures are also evaluated and shown in Fig 12. There is a positive linear relationship between the porosity increment and treatment temperatures of rock salt. The increment percentages of porosity increase from 10.96% to 58.97% with an increase in temperature from 50°C to 250°C. It is obviously seen that the porosity increased with the treatment temperature due to the damage to the internal structure of rock salt with increasing temperature.

## Discussion

Considering the results obtained from both previous studies and this study, it is clearly revealed that there are inconsistencies between the effects of temperature on the geomechanical properties of the thermally treated samples and that of the samples subjected to the tests under heating temperatures. Phatthaisong et al. [37], pointed out that the thermal effect reduced the compressive strengths of Maha Sarakham rock salt which was tested under target temperature by a heating system implemented to test setup. In line with the results of Phatthaisong et al. [37], Sriapai et al. [15] and Li et al. [14] also found that there is a negatively proportional relationship between uniaxial compressive strength and the sample temperature. On the contrary, Liang et al. [13] determined that the uniaxial compressive strength of thenardite rock salt samples, which were tested immediately after coming out of the oven, increased with the temperature and the uniaxial compressive strength at 120°C was 74.5% more than the strength determined for 20°C. Similar to the findings of Liang et al. [13], it is found that the σ_c_ of Çankırı rock salt linearly increased with the temperature in this study. These differences in the results of the studies on the thermal effects on geomechanical properties of rock salts may probably be due to the heating processes and testing procedures as well as healing behavior, rock salt types (pure, interbedded, one crystalline, polycrystalline, etc.) and various specimens shape (cylindrical, cubical, etc.). The cracks and permanent deformations occurred because of the weakening of grain-grain bonding with the increment of temperature as detected from the X-ray micro-CT images of thermally treated Çankırı rock salt. Therefore, the uniaxial compressive strength of the thermally treated such kind of rock samples quite likely decreases with temperature. On the other hand, the cracks in the internal structure of rock salt can be reduced or recovered in time by self-healing characteristics under appropriate temperature, pressure and humidity conditions. Thus, the elapsed time between the thermal treatment of samples and testing, and the environmental conditions have also an effect on the uniaxial compressive strength.

According to the stress-strain curves in Fig 8, the rock salt is becoming more ductile at a higher temperature of samples. In this study, the amount of axial strain measured under the same stress levels increased with the increasing temperature. Such that the axial strain determined at the peak stress is 2.96% at 20°C whereas it reaches up to 6.29% at 250°C. It is concluded that there is an evident correlation between ductility and the temperature of samples. It is clearly indicated that the Çankırı rock salt tends to exhibit more ductile behavior with increasing temperature when the samples are subjected to the tests under heating temperatures. Thus, the peak strengths of heated samples are evidently higher than that of the non-heated samples due to the augmented ductility (see Figs 7 and 10). That result is also compatible with the findings of Liang et al. [13] and Malkowski et al. [38].

The crack accumulation behavior and failure mechanism of rock salt under stress are evidently different from the brittle rocks (granite, sandstone, marble etc.) studied by Ohnaka and Mogi [34], Eberhardt et al. [39], Diederichs et al. [17], Tuncay and Ulusay [40], Zang et al. [41], Lei [42], Zhao et al. [43]. In brittle rocks, there is a silence period in which AE count cannot be measured whereas the AE activity starts in rock salts right after loading. Lavrov et al. [44], Zhang et al. [25], Shkuratnik et al. [45] recorded AE counts as soon as applying the axial loads on rock salt samples.

Zhang et al. [25] explained this behavior with rapidly developing bonding breakage, crystal friction, slippage and dislocation at the early stages of loading due to the weak bonding between the grains of rock salt. In this study, the AE activity generated immediately after the applying axial load to the Çankırı rock salt sample in line with the results of Lavrov et al. [44], Shkuratnik et al. [45], Zhang et al. [25]. While the AE count increases continuously in the early stages of loading, the increment rate of the AE count decreases as approaches the peak stresses (see Fig 10A2-10F2). This situation explained by Zhang et al. [25] that the energy transferred to the sample during loading is consumed in the crack evolution, as more deformation develops in rock salt than in brittle rocks. Distinctively from the studies of Lavrov et al. [44] and Zhang et al. [25], the effects of sample temperature on the AE activity of rock salt was investigated in this study. It is concluded that the crack accumulation behavior of rock salt is considerably sensitive to sample temperature due to increasing ductility with temperature. For instance, the number of AE counts recorded for 5 MPa stress level is 39 at 20°C whereas it is 108 at 250°C (see Fig 10).

**Fig 13:**
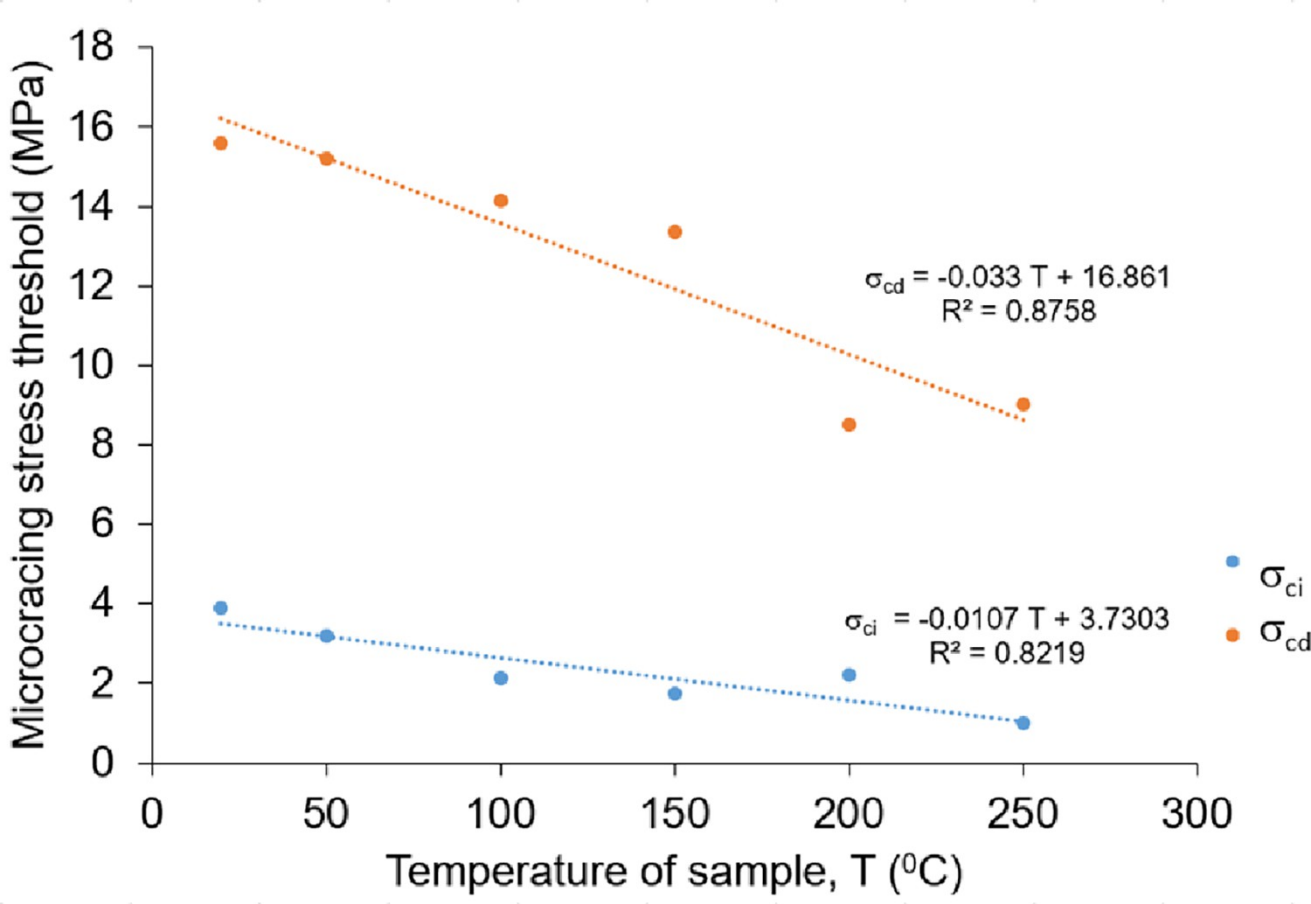
Microcracking stress thresholds (F073_ci_, F073_cd_) versus temperatures of samples.

To identify the effect of temperature on the σ_ci_ and σ_cd_ values of Çankırı rock salt, the relationships between these parameters and temperature were plotted in Fig 13. Accordingly, there are inverse linear relationships between temperature and both the σci—σ_cd_ thresholds. The σ_ci_ decreased from 3.9 MPa to 0.98 MPa with a decrement percentage of 74.9% for the temperatures 20°C and 250°C, respectively. Similarly, the σ_cd_ decreased from 15.6 MPa to 9 MPa with a decrement percentage of 42.5% for the same temperature range. The stress-strain curves of Çankırı rock salt show that plastic deformation occurs at low stress levels as the temperature of samples increases (Fig 10A1-10F1). Consequently, the cracking process of Çankırı rock salt initiates at the earlier phase of the loading with the increasing temperature.

The V_p_ of the Çankırı rock salt decreases as the temperature of the sample increases compatible with the results of Liang et al. [13] and Chen [46]. To clearly indicate the effect of temperature on V_p_, the decreasing rates of V_p_ for each temperature level are calculated and given in Table 4. The average decreasing rate of V_p_ is 1.1% at 50°C while it reaches up to 19.6% at 250°C. In addition, the Brazilian tensile strength of Çankırı rock salt is decreasing with increasing temperature. Similarly, Sriapai et al. [15] determined an inverse relationship between the Brazilian tensile strength and the temperature.

**Table 4:** The decreasing rates of V_p_ with temperature.

Temperature (°C)	20	50	100	150	200	250
Avarage Vp decrease ratio (%)	0	1.1	8.0	11.3	16.8	19.6

Microfractures and cracks in rocks are directly related to the porosity of the rocks. The augmentation of cracks observed in 3D X-ray micro-CT images of Çankırı rock salt is also an indicator of increasing values of porosity with increasing temperatures. the increment ratio of porosity of the thermally treated samples at 250°C reaches up to 58.97% because of thermal deformation. Accordingly, the porosity of rock salts is highly sensitive to change in temperature. Pan et al. [47] pointed out that the V_p_ decreases with increased porosity. Hence, the porosity may be extrapolated by determining changes in V_p_ values [47].

## Conclusions

In this study, a comprehensive experimental program was conducted to evaluate the thermal effect on the P-wave velocity (V_p_), Brazilian tensile strength (σ_t_), uniaxial compression strength (σ_c_), crack accumulation characteristics, microstructure, and porosity of Çankırı Rock Salt for the temperatures changing between 20°C and 250°C. Based on the results, the following conclusions can be summarized as follows:

1. The σ_c_ of the Çankırı rock salt increases linearly whereas the V_p_ and the σ_t_ decrease with the increasing temperature of the sample. While there is a non-linear inverse relationship between the σ_t_ and temperature of samples, the relationship between the V_p_ and temperature is linearly decreasing.
2. The ductility of rock salt tends to increase and the stress-strain curves shifted to the right while the temperature of samples increases. The amount of axial strains measured in rock salt samples tested at 250°C is higher than that of samples at 20°C.
3. Different from brittle rocks, the AE activity of rock salt appears at early stages of loading during the uniaxial compression tests. The accumulation of AE is prominently augmented with the increasing temperature of samples. Consequently, both the σci—σ_cd_ thresholds decreased with increasing temperatures of Çankırı rock salt.
4. The amount of micro and macro cracks in thermally treated rock salt samples increased significantly as detected from X-ray micro-CT scans. The porosity values determined from X-ray micro-CT analyses increase significantly with the increasing treatment temperature.
